# An Unenclosed Quasi-Static Cavity Resonator-Based Ubiquitous 3-D Wireless Power Transfer System Supporting Simultaneous Through-Wall Wireless Communications

**DOI:** 10.3390/mi16010013

**Published:** 2024-12-26

**Authors:** Qiaoli Zhang, Lingao Fan, Fangcheng Ren, Zhen Yue, Deshuang Zhao, Shuai Ding, Bingzhong Wang

**Affiliations:** 1 School of Physics, University of Electronic Science and Technology of China, Chengdu 611731, Chinabzwang@uestc.edu.cn (B.W.); 2 School of Electronic Science and Engineering, University of Electronic Science and Technology of China, Chengdu 611731, China

**Keywords:** cavity resonance wireless power transfer, quasi-static cavity resonator, 3-D, through-wall wireless communications

## Abstract

With the emergence of the Internet of Things (IoT), the demand on the wireless power supply to consumer electronics simultaneously requires much more location freedom, ease of use, and performance with wireless communications. In this paper, an unenclosed quasi-static cavity resonator (QSCR) constructed with metallic strips and the design method are proposed and theoretically analyzed. This unenclosed QSCR has a simple structure, which benefits the wireless charging for portable/wearable electronics and smart appliances in the office and home environment. Meanwhile, it can achieve simultaneous ubiquitous 3-dimensional (3-D) wireless power transfer (WPT) inside the cavity and through-wall wireless communications with external electronic devices. Simulation and experimentation are performed to verify the theoretical analysis of the proposed cavity resonator and the WPT system based on it. As demonstrated, at a powering frequency of 6.78 MHz, the unenclosed QSCR can wirelessly transfer power to the receivers with a maximum power transfer efficiency of 90.5%, and an efficiency exceeding 51.5% is obtained at almost any position within the cavity space. The measured through-wall wireless communication channel attenuation introduced by the unenclosed QSCR is below 2.87 dB. By adjusting the inserted lumped capacitor value, the system can work at any desired frequency.

## 1. Introduction

With the wide usage of electronic devices in daily life and industry, the demand on the power supply for portable consumer electronics, wearable electronics, and biomedical implants requires more energy efficiency, a smaller system size, and sufficient location freedom. As a technology of cordless energy transfer [1], wireless power transfer (WPT) can replace wired connections or battery charging, so it better satisfies the requirement of modern electronic equipment with strong mobile flexibility. Traditional inductively coupled power transfer (ICPT) and WPT based on magnetic resonance coupling (MRC) is a promising method to charge low-power consumer and household electronic devices [2]. However, these technologies using transmitter and receiver coils are typically limited to one-dimensional (1-D) or two-dimensional (2-D) configurations [3,4], which have limitations on the power distance and requirements for precise positioning and alignment. Microwave power transfer (MPT) can transfer power over long distances, but due to health and safety concerns, it is not suitable for the charging of the portable devices in indoor environments that people interact with in daily life. Recently, a few three-dimensional (3-D) WPT systems have been implemented by adopting current modulation or special transmitter shapes to reduce the system’s sensitivity to the rotation angle and misalignment of the receiver, presenting great potential for wireless charging moving objects in indoor environment. However, these 3-D transmitters often have complex structures requiring multiple coils and power sources, nonidentical currents, and an additional control circuit, which may lead to an increase in system cost and complexity [5,6,7,8,9,10,11].

At present, a cavity resonance wireless power transfer (CR WPT) technique has been proposed as a promising approach for wireless charging in a confined 3-D space [12,13,14,15]. CR WPT can transfer power by exciting cavity resonant modes inside an enclosed or semi-enclosed metallic cavity and subsequently coupling the energy from the cavity to the receivers in it. Compared with other MRC WPT technologies, CR WPT can achieve high power transfer efficiency over a relatively long transmission distance and has high insensitivity to misalignment. To realize wireless power and information transfer (WPIT) in a closed space, the metal mesh frameworks, which work as frequency selected surfaces (FSSs), were utilized to achieve a safe equipment diagnosis for greenhouse or plant facilities [16,17,18,19,20,21]. Additionally, in 2021, we proposed a wall-meshed cavity resonator constructed from the meshed metallic walls to achieve WPT without blocking wireless communications with the outside world [22].

A ubiquitous CR WPT using a quasi-static cavity resonator (QSCR) was proposed in [23]. A room-wide wireless charging and load-modulation communication system was demonstrated to validate the possibility of seamless WPT to receivers placed anywhere in the indoor spaces [24]. Furthermore, the utilization of multimode QSCR can address the challenges of the necessity of the center pole and null zone of power delivery [25,26]. Compared to conventional CR WPT, QSCR WPT has the advantage of confining the electric field, which mainly interferes with biological tissues, within the lumped capacitors. However, most of the quasi-static cavities for CR WPT are fully enclosed structures composed of closed metallic walls. In practical applications, it is costly and inconvenient to construct an all-metal enclosed cavity. Moreover, the communication between external signals and devices inside the cavity resonator will be hindered by the electromagnetic shielding effect of the enclosed metal structure.

To address the limitations of the traditional QSCR on blocking wireless communications and to satisfy the 3-D power supply requirements of mass personal and smart home electronic devices, a CR WPT system based on the unenclosed QSCR is proposed in this paper. By exciting the unenclosed QSCR, the quasi-static toroidal magnetic field is generated, which can transfer power to the receivers at most positions inside the cavity through magnetic resonance coupling. In addition, due to the unenclosed structure, the proposed cavity resonator will not block the through-wall wireless communications with the outside world. Thus, this WPT technique can effectively solve the power supply problem for dozens or hundreds of Internet of Things (IoT) electronic devices in smart homes or offices.

## 2. Theory of Operation and Analytical Modeling

### 2.1. Unenclosed Quasi-Static Cavity Resonator

**Figure 1:**
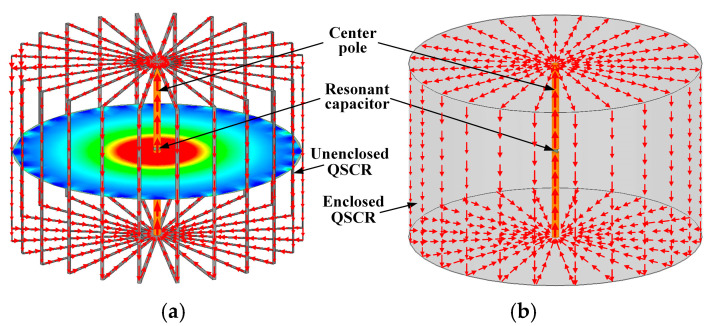
(**a**) Configuration with the surface current distribution on the wall of the unenclosed quasi-static cavity resonator (QSCR) and magnetic field distribution inside it; (**b**) surface current distribution on the wall of the enclosed QSCR [23].

Figure 1a shows the model of an unenclosed QSCR, which is composed of several C-shaped metallic strips, and a conductive pole is connected to the metallic strips at the center of the cavity. The material of the metallic strips can be any metals with excellent conductivity, and in applications, they can be replaced with cable lines. The unenclosed QSCR can be of any shape, such as rectangular, cylindrical, or spherical shape. Here, for fabrication simplicity, we design the unenclosed QSCR with a cylindrical shape. To generate resonance, a resonant capacitor is inserted across a gap in the center pole. By using a driver coil to excite the unenclosed QSCR, induced currents can be generated on the surface of the metallic strips, which flow through the center pole, which are shown as the arrows in Figure 1a. The current that flows through each metallic strip is almost the same, whereas the quasi-static magnetic field generated in the cavity is annular around the center pole. For comparison, Figure 1b shows the current distribution on the wall of the enclosed QSCR [23], which is almost identical to that in the unenclosed QSCR.

Since the difference between the length and the width of the metallic strip is significant, ignoring the width, the induced line current on the *n*th metallic strip is *I_n_* (*n* = 1, 2, …, *N*), where *N* is the number of metallic strips, and the angle between two metallic strips is *θ* = 2*π*/*N*. Assuming that *N* line currents have identical intensities, the current on the center pole *I_total_* can be written as

(1) $$I_{total}=\sum_{n=1}^{N}I_{n}=N\cdot I_{n}.$$

**Figure 2:**
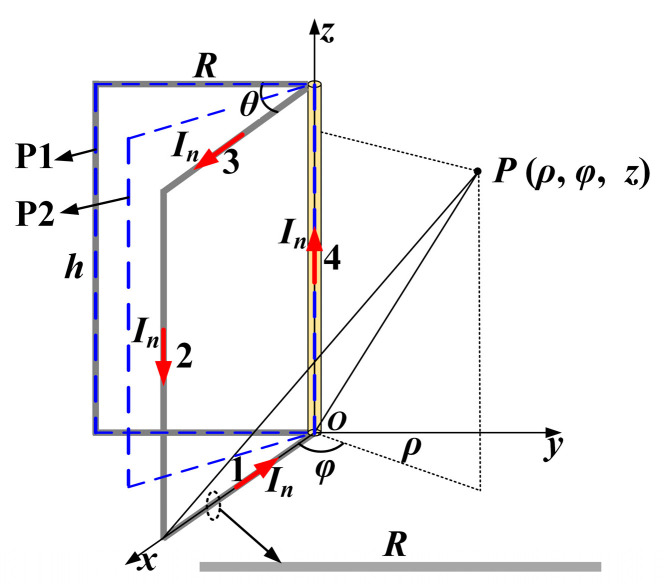
Model of a unit of the unenclosed quasi-static cavity resonator (QSCR).

The magnetic field distribution in the unenclosed QSCR is generated by *N* line currents *I_n_*, each of which can be considered as a combination of four connected line currents, as shown in Figure 2. P1 is the plane where the metallic strip is located, and P2 is the angle-bisecting plane of two adjacent metallic strips.

(2) $$\left\{\begin{array}{c} H_{n,1\varphi }=-\frac{I_{n}z\cos\left(\varphi +(n-1)\theta \right)}{4\pi \left[\rho^{2}\sin^{2}\left(\varphi +(n-1)\theta \right)+z^{2}\right]}\left[\frac{\rho \cos\left(\varphi +(n-1)\theta \right)}{\sqrt{\rho^{2}+z^{2}}}+\frac{R-\rho \cos\left(\varphi +(n-1)\theta \right)}{\sqrt{R^{2}+\rho^{2}+z^{2}-2R\rho \cos\left(\varphi +(n-1)\theta \right)}}\right]\\ H_{n,2\varphi }=-\frac{I_{n}(h-z)\cos\left(\varphi +(n-1)\theta \right)}{4\pi \left[\rho^{2}\sin^{2}\left(\varphi +(n-1)\theta \right)+{\left(h-z\right)}^{2}\right]}\left[\begin{array}{c} \frac{\rho \cos\left(\varphi +(n-1)\theta \right)}{\sqrt{\rho^{2}+{\left(h-z\right)}^{2}}}+\\ \frac{R-\rho \cos\left(\varphi +(n-1)\theta \right)}{\sqrt{R^{2}+\rho^{2}+{\left(h-z\right)}^{2}-2R\rho \cos\left(\varphi +(n-1)\theta \right)}} \end{array}\right]\\ H_{n,3\varphi }=-\frac{I_{n}\left[R\cos\left(\varphi +(n-1)\theta \right)-\rho \right]}{4\pi \left[R^{2}+\rho^{2}-2R\rho \cos\left(\varphi +(n-1)\theta \right)\right]}\left[\begin{array}{c} \frac{z}{\sqrt{R^{2}+\rho^{2}+z^{2}-2R\rho \cos\left(\varphi +(n-1)\theta \right)}}\\ +\frac{h-z}{\sqrt{R^{2}+h^{2}+\rho^{2}+z^{2}-2R\rho \cos\left(\varphi +(n-1)\theta \right)-2hz}} \end{array}\right]\\ H_{n,4\varphi }=-\frac{I_{n}}{4\pi \rho }\left(\frac{h-z}{\sqrt{h^{2}+\rho^{2}+z^{2}-2hz}}+\frac{z}{\sqrt{\rho^{2}+z^{2}}}\right) \end{array}\right.\;\;\;\;n=1,\;2,\cdots N.$$

Compared with the magnetic field strength in the *φ* direction, the components in the *ρ* and *z* directions can be negligible. Thus, assuming that the axis of the receiver coil is along the *φ* direction, it is only necessary to consider the magnetic field intensity in the *φ* direction. For any position *P*(*ρ*, *φ*, *z*) inside the cavity, the corresponding magnetic induction intensities in the *φ* direction generated by line currents 1–4 can be written as Equation (2), where *h* and *R* are the height and radius of the unenclosed QSCR, respectively. Thus, the total magnetic field intensity in the *φ* direction at position *P*(*ρ*, *φ*, *z*) can be written as follows:

(3) $$H_{\varphi }=\sum_{n=1}^{N}-\left[H_{n,1\varphi }+H_{n,2\varphi }+H_{n,3\varphi }+H_{n,4\varphi }\right].$$

**Figure 3:**
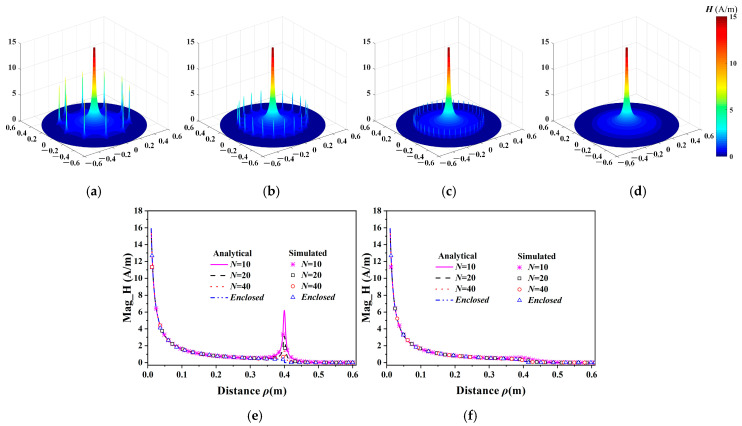
Analytical magnetic field intensities in the unenclosed QSCR with different numbers of metallic strips (**a**–**d**), and lineslices on planes P1 (**e**) and P2 (**f**).

By setting a 1-A current source, Figure 3 shows the magnetic field intensities in the unenclosed QSCR analyzed using Equations (1)–(3) and the full-wave simulated results based on the finite element method (FEM) for various numbers of metallic strips. The analytical results are consistent with the simulated ones, and the small discrepancy near the metallic strip on plane P1 is due to the lack of position accuracy in simulations. Meanwhile, the quasi-static magnetic fields ***H****_φ_* on planes P1 and P2 decay with distance *ρ* except for a peak near each metallic strip on plane P1. These magnetic field peaks are introduced by the currents on the metallic strips at *ρ* = 40 cm, which introduce a localized magnetic field around each metallic strip. Moreover, with more metallic strips, the magnetic field intensity for the unenclosed QSCR is very close to the enclosed one, which implies that adopting many more metallic strips is beneficial to the WPT, but impedes the passage of wireless communication signals.

**Figure 4:**
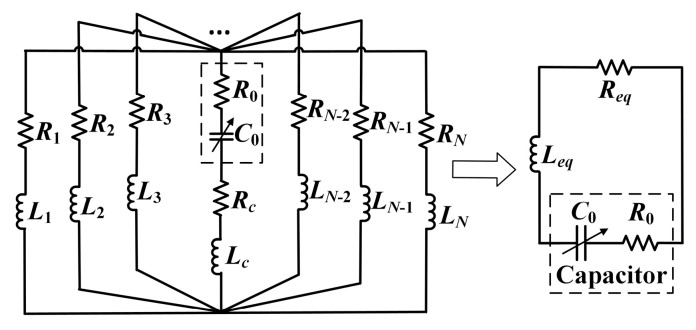
Equivalent circuit model of the unenclosed quasi-static cavity resonator (QSCR).

Figure 4 shows the equivalent circuit model of the unenclosed QSCR. *L_eq_* and *R_eq_* are the equivalent inductance and resistance of the cavity, respectively. The equivalent inductances and resistances of the nth metallic strip and center pole *L_n_*, *R_n_*, *L_c_*, and *R_c_* can be determined using Equations (4)–(7) [27,28]. With the capacitor across the gap of the center pole, multiple RLC resonance circuits can be formed.

(4) $$L_{n}=\frac{\mu_{0}}{2\pi }l\left[\ln\left(\frac{2l}{w+t}\right)+0.5+0.2235\frac{w+t}{l}\right],$$

(5) $$R_{n}=\frac{663l\sqrt{f_{0}}\cdot 10^{-10}}{2(w+t)}\cdot \frac{w}{t},$$

(6) $$L_{c}=\frac{\mu_{0}}{2\pi }h\left[\ln\left(\frac{2h}{r}\right)-1\right],$$

(7) $$R_{c}=\frac{h}{2r}\sqrt{\frac{\mu }{\pi \sigma }}\sqrt{f_{0}},$$

(8) $$L_{eq}=\frac{1}{\sum_{n=1}^{N}\frac{1}{L_{n}}}+L_{c},\;\;R_{eq}=\frac{1}{\sum_{n=1}^{N}\frac{1}{R_{n}}}+R_{c},$$

where *l* = 2*R* + *h*, *w*, and *t* are the total length, width, and thickness of the metallic strip, respectively; *μ*_0_ is the permeability of free space; and *h*, *r*, *μ*, and *σ* are the height, radius, permeability, and conductivity of the center conductive pole, respectively.

When *h* = 50 cm, *R* = 40 cm, *w* = 5 mm, *t* = 2 mm, and *r* = 10 mm, *L_eq_* and *R_eq_* are 360 nH and 13.7 mΩ, respectively. Thus, the resonant frequency *f*_0_ of the unenclosed QSCR is determined as follows [12]:

(9) $$f_{0}=\frac{1}{2\pi \sqrt{L_{eq}C_{0}}}.$$

Its quality factor *Q_q_* is given by

(10) $$Q_{q}=\frac{2\pi f_{0}L_{eq}}{R_{eq}+R_{0}}.$$

By inserting a capacitor with capacitance *C*_0_ = 1.53 nF and equivalent series resistance *R*_0_ = 11.8 mΩ across the gap of the center pole, we obtain *f*_0_ and *Q_q_* to be 6.78 MHz and 602, respectively, whereas the simulated and measured *Q_q_* at 6.78 MHz are 656 and 588, respectively [29]. As presented in Equation (9), the working frequency of the proposed unenclosed QSCR can be adjusted by tuning the inserted capacitance, which helps debug the system and perform the frequency-reconfigurable WPT system. Meanwhile, its simple structure and low cost make it promising in wireless power supply for 3-D spaces, such as smart homes and smart offices.

### 2.2. Unenclosed QSCR-Based WPT System

**Figure 5:**
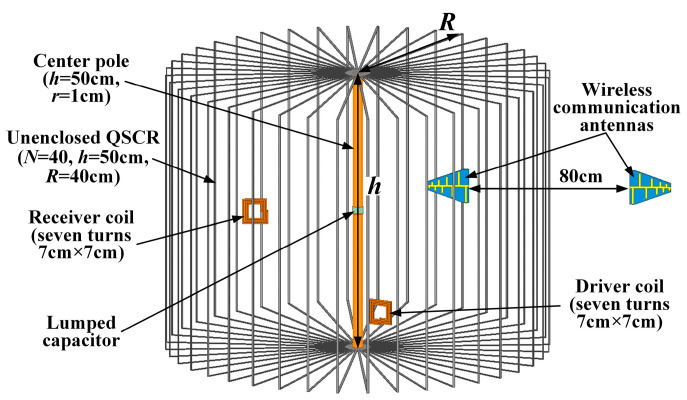
Configuration of the unenclosed quasi-static cavity resonator (QSCR)-based WPT and through-wall wireless communication system.

Figure 5 shows the configuration of the unenclosed QSCR-based WPT and through-wall wireless communication system. For WPT, two seven-turn 7 cm × 7 cm square copper coils are used as the driver and receiver coils. The resonant mode is stimulated by the driver coil, and the energy is inductively coupled from the power source into the resonator cavity. For through-wall wireless communication, a wireless communication system is established using a pair of log-periodic antennas, which are placed inside or outside the unenclosed QSCR.

According to the coupled-mode theory [12], the WPT efficiency mainly depends on the coupling coefficient *κ_qr_* between the unenclosed QSCR and the receiver coil, which is given by

(11) $$\kappa_{qr}=\frac{1}{4}\frac{\omega_{0}\beta }{L_{r}\alpha^{1/2}\zeta }=\frac{\sqrt{2}}{4}\frac{\omega_{0}\beta }{\sqrt{L_{r}\alpha }},$$

where *ω*_0_ is the angular resonant frequency of the unenclosed QSCR; *α* is the total magnetic energy stored in the cavity; *β* is the magnetic flux that interlinks with the receiver coil; and *L_r_* and *ζ* are the inductance of the receiver coil and a constant related to the energy storage, respectively [12].

(12) $$\alpha =\underset{V}{∭}\frac{\mu_{0}}{2}{\left|\vec{H}\right|}^{2}dV,$$

(13) $$\beta =\iint_{A}\mu_{0}\vec{H}\cdot \vec{n}dA.$$

Here, *V* is the entire volume of the unenclosed QSCR, $\vec{n}$ is the unit normal vector of the receiver coil surface, and *A* is the area enclosed by the receiver coil. If the size difference between receiver coil and unenclosed QSCR is significant, the magnetic flux passing through the surface of the receiver coil can be assumed to be uniform. Thus, *α* and *β* can be calculated as follows [12]:

(14) $$\alpha =\frac{1}{2}L_{eq}I_{total}^{2},$$

(15) $$\beta =\mu_{0}Hl_{r}^{2}N_{r},$$

where *l_r_* and *N_r_* are the side length and the number of turns of the square receiver coil, respectively.

With the calculated coupling coefficient *κ_qr_* and quality factors *Q_q_* and *Q_r_* of the cavity and receiver coil, the maximum WPT efficiency *η_max_* at *ω*_0_ is [12]

(16) $$\eta_{max}=\frac{\chi }{{\left(1+\sqrt{1+\chi }\right)}^{2}},$$

(17) $$\chi =\frac{4Q_{q}Q_{r}{\left|\kappa_{qr}\right|}^{2}}{\omega_{0}^{2}}.$$

**Figure 6:**
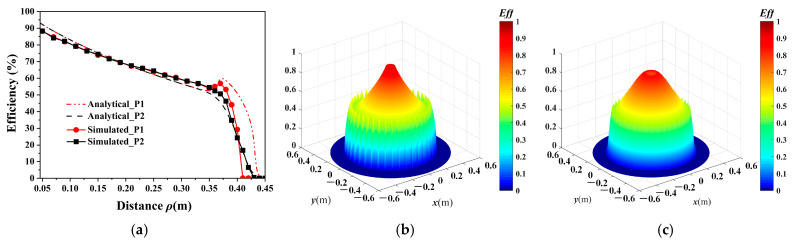
(**a**) Lineslice to compare the analytical and simulated efficiencies on planes P1 and P2. (**b**) Analytical and (**c**) simulated efficiencies over the *xy* plane at a height of *z* = 20 cm.

Using the measured *Q_q_* = 588 and *Q_r_* = 135, the WPT performance is evaluated using Equations (11)–(17) and the full-wave simulation based on FEM. For a receiver coil at a height of *z* = 20 cm, Figure 6a shows the analytical and simulated *η_max_* with the load tuned to a value that maximizes the WPT efficiency [23,29]. Here, because the magnetic field intensity decreases with the distance, Equation (13) instead of Equation (15) is used to calculate *β*. Figure 6b,c show that the efficiencies are circumferentially symmetrical and decrease with increasing distance from the center pole, which well fits the trend of the quasi-static magnetic field ***H*** in Figure 3. Considering the size of the receiver coil, the efficiencies on planes P1 and P2 within 35 cm from the center pole are evaluated, which are consistent well with each other. Thus, for simplicity, only the efficiencies on plane P1 are considered in the later study.

**Figure 7:**
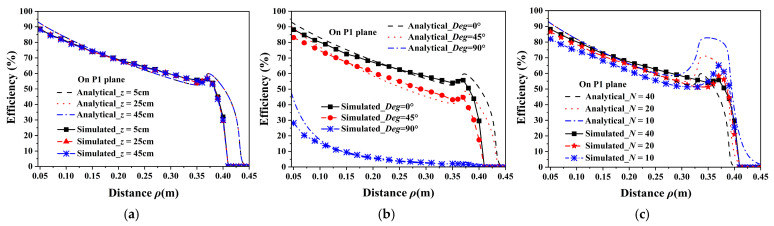
Lineslice to compare the analytical and simulated efficiencies on plane P1 for different (**a**) vertical positions of the receiver coil, (**b**) rotation angles of the receiver coil, and (**c**) numbers of metallic strips.

Figure 7 shows the comparison of the analytical and simulated efficiencies for the receiver coil on plane P1. It shows perfect stability performance when the vertical position of the receiver coil changes. Meanwhile, with increasing rotation angle, the efficiency decreases, especially at approximately 90°. The main reason is that when the receiver coil is parallel to the magnetic line of force, almost no magnetic flux penetrates the coil. It should be noted that the rotation of the receiver coil is performed around the *z*-axis in this work; for rotations around other axes, Equation (13) is also applicable. Moreover, when the number of metallic strips decreases from 40 to 20 to 10, the efficiencies slightly decrease.

According to the above analysis, the unenclosed QSCR-based WPT system presents uniform and stable wireless charging with high WPT efficiency inside the cavity. Moreover, the number of the metallic strips and the rotation angle of the receiver coil, except for some large angles, hardly affect the WPT performance.

## 3. Experiments and Results

### 3.1. WPT Performance of the Proposed System

**Figure 8:**
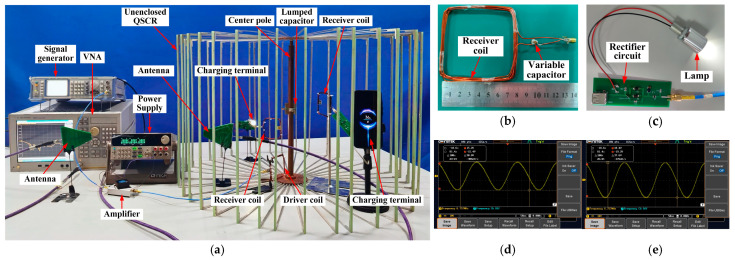
Setup of the unenclosed quasi-static cavity resonator (QSCR)-based WPT and wireless communication system. (**a**) System setup, (**b**) receiver coil, (**c**) rectifier circuit, and voltage waveforms on the receiver coil at *ρ* = 30 cm for (**d**) *R_L_* = 10 Ω and (**e**) *R_L_* = 20 Ω.

Figure 8 shows the experiment prototype of the WPT and through-wall wireless communication system based on the unenclosed QSCR with the received voltage waveforms of the WPT. The unenclosed QSCR with an 80 cm diameter and a 50 cm height is composed of 40 C-shaped metallic strips, connecting with a copper-plated pole at the center of the cavity. The metallic strips are made of aluminum (*σ* = 2.265 × 107 S/m) and reinforced with acrylic strips for firmness. Actually, the metallic strips can be replaced with cable lines, which are low cost and easier to set on the room walls, ceiling, and floor. To tune the unenclosed QSCR that resonates at 6.78 MHz, several capacitors in parallel are inserted across the gap of the center pole to achieve an equivalent capacitance value of 1446 pF. The seven-turn square coils with 7 cm width on each side are placed inside the unenclosed QSCR as the driver and receiver coils of the WPT system. In addition, for impedance matching, the driver coil is positioned at a height of 6 cm and the distance from the center pole ranges from 5 cm to 15 cm.

**Figure 9:**
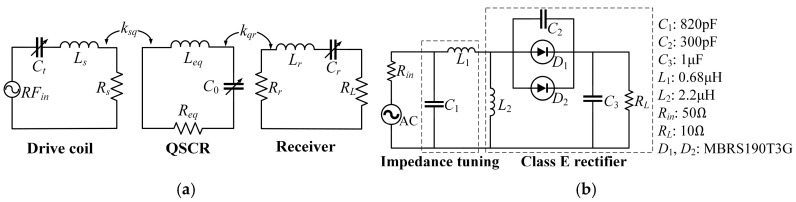
Circuit model of the (**a**) unenclosed quasi-static cavity resonator (QSCR)-based WPT system and (**b**) rectifier circuit.

As shown in Figure 9a, in the equivalent circuit model of the WPT system, *L_s_*, *R_s_*, *L_r_*, and *R_r_* are the equivalent inductances and resistances of the driver coil and receiver coil, respectively. The series variable capacitors *C_t_* and *C_r_* are connected with the coils for impedance matching (IM). Rectifier circuits in Figure 9b are attached to the receiver coils to drive a lamp and charge a mobile phone. In the measurement, the power of the signal generator with a maximum output power of 25 dBm is applied to the amplifier and excites the driver coil with 5.89 W power. Terminals such as the lamp and mobile phone receive power by coupling the receiver coils with the unenclosed QSCR.

**Figure 10:**
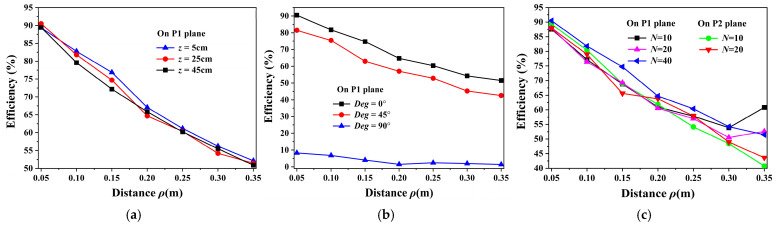
Measured WPT efficiencies (**a**) at different positions in the vertical direction, (**b**) for different rotation angles of the receiver coil, and (**c**) for different numbers of metallic strips.

Figure 10 shows the measured maximum efficiencies *η_max_* [26], which are obtained by adjusting the transmitter coil position to achieve optimal impedance matching [30]. The results in Figure 10a,b are achieved on plane P1. Thus, in practice, due to the size of the receiver coil, the extreme position to which the receiver coil can move is approximately 36 cm, which is neglected in the theoretical analysis. The performance of the WPT is evaluated with different positions and rotation angles of the receiver coil, and with different numbers of metallic strips.

(1) Position of the receiver: In Figure 10a, the measured *η_max_* remains almost stable with the change in the vertical position of the receiver coil. Hence, the charging terminal can achieve a relatively stable efficiency when vertically moving in the cavity. These results are consistent with the simulated ones in Figure 7a, except at a few positions. Furthermore, when the receiver coil is normal to the magnetic fluxes, in any position inside the cavity, the measured efficiency is more than 51.5%, and the maximum efficiency is 90.5%.
(2) Rotation angle: Figure 10b shows the tolerance of the receiver coil’s rotation angle in the proposed design. When the receiver coil is rotated from 0° to 45°, the efficiency decreases by approximately 11%, and its minimum efficiency decreases from 51.5% to 42.56%, which implies that the efficiency exceeds 42% in all powering areas. However, when the coil is rotated to 90°, the efficiency is less than 10%. Thus, this WPT system has a limitation on the direction of the receiver coil at large rotation angle, which can be solved by adopting the three orthogonal coils as the receivers.
(3) Number of metallic strips: The effect of the number of metallic strips is illustrated in Figure 10c. As expected, on both planes P1 and P2, the measured efficiencies for the unenclosed QSCR composed of 40, 20, and 10 metallic strips decrease with increasing distance and remain almost stable with fewer metallic strips. For the case of 40 metallic strips, the efficiency is slightly higher than that of the other cases due to a better adaptive impedance matching in this case.
(4) Wireless charging for multiple coils: As shown in Figure 8, the lamp and mobile phone can be simultaneously charged, and the measured WPT efficiencies for two receiver coils are presented in Table 1. *η*_1_, *η*_2_, and *η_total_* are the efficiencies with static impedance tuning (|S_21_|^2^), and *η_max_* is the total efficiency with adaptive impedance tuning for two receiver coils [30]. An efficiency up to 87.1% implies that the unenclosed QSCR can simultaneously power multiple electronic devices with high WPT efficiency.

### 3.2. Through-Wall Wireless Communication Performance of the Proposed System

**Figure 11:**
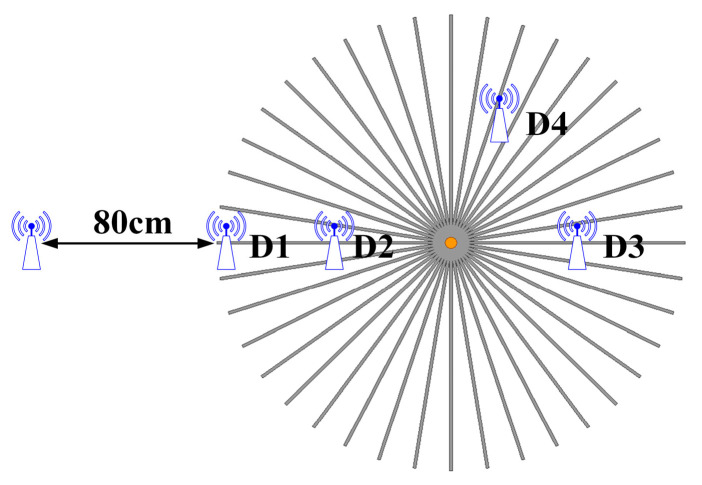
Diagram of the wireless communication measurement setup.

To validate the through-wall wireless communication performance with the simultaneous WPT capability of the proposed unenclosed resonator, a set of log-periodic antennas as wireless communication transceivers is used to build a simple communication system, as shown in Figure 8. By putting both antennas in free space, inside the unenclosed QSCR, or one outside and one inside the cavity, the wireless communication channel attenuations are measured at 2.45, 3.5, and 5.8 GHz, as shown in Table 2. In the measurement, the distance between two antennas is always maintained at 80 cm. In the case of one antenna inside and one outside, by moving the antenna inside the cavity from positions D1, D2, and D3 to D4 as shown in Figure 11, the maximum channel attenuation introduced by the unenclosed QSCR is approximately 2.87 dB at position D3. The reason may be the blocking of the center pole. For some cases such as position D1 at 5.8 GHz, the channel attenuation is smaller than that in free space because the multiple reflection of wireless signals in the cavity causes the multipath effect.

The IEEE802.15.4 RF modules CC2530 are used to measure the communication quality using an internal index link quality indicator (LQI) [31]. In free space, the LQI is 107; with one module inside and another outside the unenclosed QSCR, the LQI is approximately 95; and when both transceiver modules are inside, the LQI is up to 112. Therefore, the wireless channel loss introduced by the unenclosed QSCR is negligible, which implies that the WPT system based on the unenclosed QSCR can support ubiquitous 3-D WPT with simultaneous through-wall wireless communications.

### 3.3. SAR Assessment

**Figure 12:**
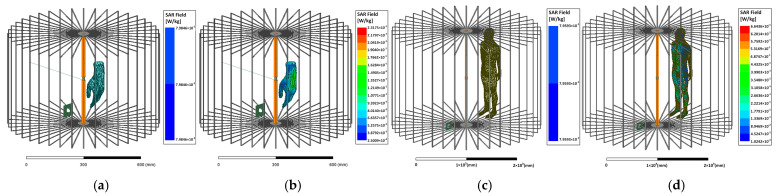
SAR of the proposed WPT system with different cavity size. (**a**,**b**) are the simulated average and localized SAR for small-size unenclosed QSCR, respectively. (**c**,**d**) are the simulated average and localized SAR for room-size unenclosed QSCR, respectively. The small-size unenclosed QSCR is with an 80 cm diameter and a 50 cm height, and the human hand model is positioned at *ρ* = 10 cm. The room-size cavity has a diameter of 1.5 m and a height of 2 m, with the human model at *ρ* = 50 cm.

The specific absorption rate (SAR) is commonly used for a quantitative assessment of the human exposure to the electromagnetic field. Considering the ICNIRP guidelines [32], which limits the average SAR to 0.08 W/kg and the localized SAR to 2 W/kg, the SAR assessments are performed on a human hand model and a 1.88 m full-body model in the small-size and room-size unenclosed QSCR, respectively. As shown in Figure 12, for the small-size unenclosed QSCR, the average SAR and localized SAR are 0.0798 W/kg and 0.23175 W/kg when the system operates at a power transfer efficiency of 50% and 14 W transmitted power. While the cavity is enlarged to room size, the average SAR and localized SAR are 0.0796 W/kg and 0.664 W/kg for 80 W transmitted power. It implies that for a large-size space, such as a room or a workshop, the safe transmitted power levels can reach up to several hundred to several thousand watts.

## 4. Comparison with Other WPT Systems

Table 3 presents the comparison of the critical specifications of the unenclosed QSCR-based WPT and through-wall wireless communication system with other related works. The cavity in [15] was designed with slots on the walls for freely moving animal experiments, but it has low power transfer efficiency and internal communication within the cavity. Compared to the systems in [16,17,18,19,20,21,22], the unenclosed QSCR-based system has the lowest wireless communication attenuation, the highest maximum wireless power transfer efficiency of approximately 90.5%, a relative uniform efficiency distribution throughout the entire cavity, and frequency tuning ability. Compared to the enclosed QSCR WPT system in [26], the unenclosed QSCR has good efficiency uniformity, low cost, easy construction, and the ability to support wireless communication with outside electronic devices. Its benefits in communication performance, system cost, and scalability in the frequency, volume, power, and number of charged devices make it more suitable for meeting the demand for powering mass household consumer and wearable electronics in the IoT era.

## 5. Conclusions

In this paper, an unenclosed QSCR with a simple configuration was proposed, which was constructed from 40 C-shaped metallic strips and a center conductive pole. The field distribution and operation principle of the proposed cavity resonator were investigated using the coupled mode theory and circuit model analysis. The CR WPT system based on the unenclosed QSCR can achieve ubiquitous 3-D WPT and support simultaneous through-wall wireless communications between inside and outside electronic devices. Simulations and experiments were performed to validate the WPT and through-wall wireless communication performance. The measured results show that the proposed CR WPT system achieves a maximum power transfer efficiency of approximately 90.5%, with simultaneous 2.87 dB extra wireless communication channel attenuation introduced by the unenclosed QSCR. Furthermore, for a receiver coil vertical to the magnetic field, the system demonstrated excellent charging coverage performance, with an efficiency exceeding 51.5% in all areas of the cavity space. The proposed unenclosed QSCR-based WPT method has great potential in practical WPT applications in ubiquitous 3-D wireless charging for small household and office electronic devices and wireless remote control outside the charging area.

## Figures and Tables

**Table 1:** Wireless power transfer performance with two receiver coils.

*ρ* of Rx1 (*φ* = 0°, *z* = 15 cm)	*ρ* of Rx2 (*φ* = 90°, *z* = 35 cm)	*η*_1_ (%)	*η*_2_ (%)	*η_total_* (%)	*η_max_* (%)
10 cm	10 cm	41.1	37.9	79	87.1
10 cm	20 cm	48.4	27.7	76.1	85.9
10 cm	30 cm	52.5	20.3	72.8	85.5
20 cm	20 cm	34.9	32.6	67.5	78.1
20 cm	30 cm	39.1	26.3	65.4	75.6
30 cm	30 cm	28.6	31.2	59.8	70.2

**Table 2:** Wireless communication attenuation introduced by the unenclosed QSCR.

Freq. (GHz)	Both in Free Space (dB)	Both Inside Unenclosed QSCR (dB) *	One Inside and One Outside *			
			D1 (dB)	D2 (dB)	D3 (dB)	D4 (dB)
2.45	25.89	+1.97	+0.11	+0.05	+2.21	−0.10
3.5	24.9	+2.17	+0.20	+0.69	+1.53	+0.71
5.8	37.6	−1.84	−0.90	−0.03	−2.87	+0.65

* The attenuation values for the situations with both antennas inside the unenclosed QSCR and with one inside and one outside the unenclosed QSCR are the incremental or decremental losses introduced only by the unenclosed QSCR, which is compared with the case of both in free space.

**Table 3:** Comparison of the reported CR WPT systems.

Ref.	Freq. (MHz)	Cavity Size (mm)	Cavity Type	Receiver Size (cm)	*η_max_*/*η_min_*	Positions Exceeding 50% Efficiency	Freq. Tuning	Attenuation of Wireless Communication *
[14]	347.99	60.96 × 60.96 × 30	Enclosed with slots	Coil/0.8 × 0.8	14.32%/0.3%	N/A	No	N/A
[15,16,17,18]	450	47.3 × 47 × 80	Meshed	Probe/8.3	65.7%/26.9%	N/A	No	N/A
[19,20]	357	49.6 × 47.7 × 29.1	Meshed on a wall	Probe/10	74.4%/32.4%	N/A	No	11.9 dB @2.425 GHz
[21]	278	91.6 × 91.6 × 120.4	Meshed	Coil/4 × 4	85.3%/30%	70%	No	5.1 dB @2.45 GHz
[25]	1.20/1.34	300 × 300 × 200	Enclosed QSCR	Coil/15 × 15	95%/37.1%	98%	Yes	N/A
This work	6.78	*R* = 40, *H* = 50	Unenclosed QSCR	Coil/7 × 7	90.5%/51.5%	100%	Yes	2.87 dB @5.8 GHz

* Attenuation of wireless communication signals through the wall of the unenclosed QSCR. N/A = not applicable.

## Data Availability

Data are contained within the article.
